# Provoked versus spontaneous migraine attacks: pathophysiological similarities and differences

**DOI:** 10.1186/s10194-022-01464-2

**Published:** 2022-07-23

**Authors:** Håkan Ashina, Rune Häckert Christensen, Messoud Ashina

**Affiliations:** 1 Danish Headache Center, Department of Neurology, Rigshospitalet Glostrup, Faculty of Health and Medical Sciences, University of Copenhagen, Valdemar Hansens Vej 5, 2600 Glostrup, Copenhagen, Denmark; 2 Department of Neurorehabilitation / Traumatic Brain Injury, Rigshospitalet, Copenhagen, Denmark

**Keywords:** Trigeminovascular System, Headache, Aura, Premonitory Symptoms, Trigger Factors

## Abstract

**Background:**

The onset and duration of spontaneous migraine attacks are most often difficult to predict which, in turn, makes it challenging to study the neurobiologic underpinnings of the disease in a controlled experimental setting. To address this challenge, human provocation studies can be used to identify signaling molecules (e.g. calcitonin gene-related peptide, pituitary adenylate cyclase-activating polypeptide) that, upon intravenous or oral administration, induce migraine attacks in people with migraine and mild or no headache in healthy volunteers. This approach has proven to be valid for decades and plays an integral role in mapping signaling pathways underlying migraine pathogenesis and identification of novel drug targets. However, the question arises as to whether the pathogenic mechanisms of provoked and spontaneous migraine attacks differ. In this paper, we provide an opinionated discussion on the similarities and differences between provoked and spontaneous attacks based on the current understanding of migraine pathogenesis.

**Methods:**

The PubMed database was searched in July 2022 for original research articles on human provocation studies that included participants with migraine. The reference lists of originally identified articles were also searched and we selected those we judged relevant.

**Discussion:**

People with migraine describe that provoked attacks resemble their spontaneous attacks and can be treated with their usual rescue medication. From a neurobiologic standpoint, provoked and spontaneous migraine attacks appear to be similar, except for the source of migraine-inducing substances (exogenous vs. endogenous source). In addition, provoked attacks can likely not be used to study the events that precede the release of migraine-inducing signaling molecules from sensory afferents and/or parasympathetic efferents during spontaneous attacks.

Migraine is a disabling neurovascular disorder that is characterized by recurrent headache attacks and accompanying symptoms, such as nausea, photo-, and phonophobia [1]. The onset and duration of spontaneous migraine attacks are often unpredictable which, in turn, makes it difficult to study its neurobiologic underpinnings in a controlled experimental setting [2]. This has made the use of human provocation studies valuable for researchers who aim to identify migraine-inducing substances and map signaling pathways that are responsible for migraine pathogenesis [3]. The experimental set-up most often includes a randomized, double-blind, placebo-controlled, 2-way crossover design, in which study participants are allocated to receive administration of a hypothesized migraine-inducing substance or placebo on two experimental days that are separated by a wash-out period of at least seven days [2, 3]. The general idea is that healthy volunteers develop no more than a mild headache, whereas people with migraine develop migraine attacks [2, 3]. This principle has proven to be valid through decades of research and continues to play an important role in the discovery of novel drug targets for migraine and other headache disorders [2, 3]. However, there remains a point of scientific contention among researchers. Are the pathogenic mechanisms of provoked migraine attacks similar or different from spontaneous migraine attacks? We aim to shed light on this issue, with an opinionated discussion on lessons learned and outstanding research questions.

## Methods

The PubMed database was searched in July 2022 for original research articles on human provocation studies that included participants with migraine. We searched for “migraine” in combination with the terms “adrenomedullin”, “amylin”, “calcitonin gene-related peptide”, “glyceryl trinitrate”, “nitric oxide”, “nitroglycerin”, “phosphodiesterase inhibitor”, “pituitary adenylate cyclase-activating polypeptide”, “potassium channel”, “prostaglandins”, and “vasoactive intestinal polypeptide”. We also searched the reference lists of originally identified articles and selected those we deemed relevant.

## Lessons learned and outstanding research questions

**Fig. 1:**
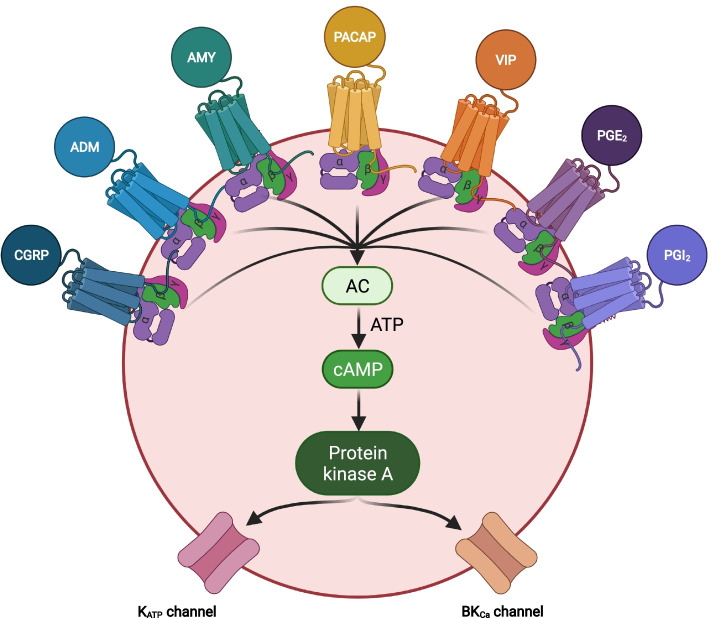
cAMP-dependent pathways in migraine pathophysiology. The cell is a vascular smooth muscle cell within the walls of intracranial arteries. Experimental studies have shown that binding of calcitonin gene-related peptide (CGRP), adrenomedullin (ADM), amylin (AMY), Pituitary adenylate cyclase-activating polypeptide (PACAP), vasoactive intestinal polypeptide (VIP), prostaglandin E_2_ (PGE_2_), and prostaglandin I_2_ (PGI_2_) to their G protein-coupled receptors increases the intracellular concentration of cyclic adenosine monophosphate (cAMP) and thereby activates the cAMP-dependent pathway. This will then activate protein kinase A which, in turn, results in outflow of potassium via opening of adenosine triphosphate-sensitive potassium (K_ATP_) channels and large conductance calcium-activated potassium (BK_Ca_) channels. The end result is hyperpolarization of the vascular smooth muscle and accompanying vasodilation which is hypothesized to provide the necessary chemical and mechanical stimuli needed to activate and sensitize perivascular nociceptors [2]. AC, adenylate cyclase; ADM, adrenomedullin; AMY, amylin; ATP, adenosine triphosphate; BK_CA_, large conductance calcium-activated potassium channels; cAMP, cyclic adenosine monophosphate; CGRP, calcitonin-gene related peptide; K_ATP_-channels, adenosine triphosphate-sensitive potassium channels; PACAP, pituitary adenylate cyclase activating polypeptide; PGE_2_, prostaglandin E_2_; PGI_2_, prostaglandin I_2_; Protein kinase A, cAMP-dependent protein kinase; VIP, vasoactive intestinal polypeptide

**Fig. 2:**
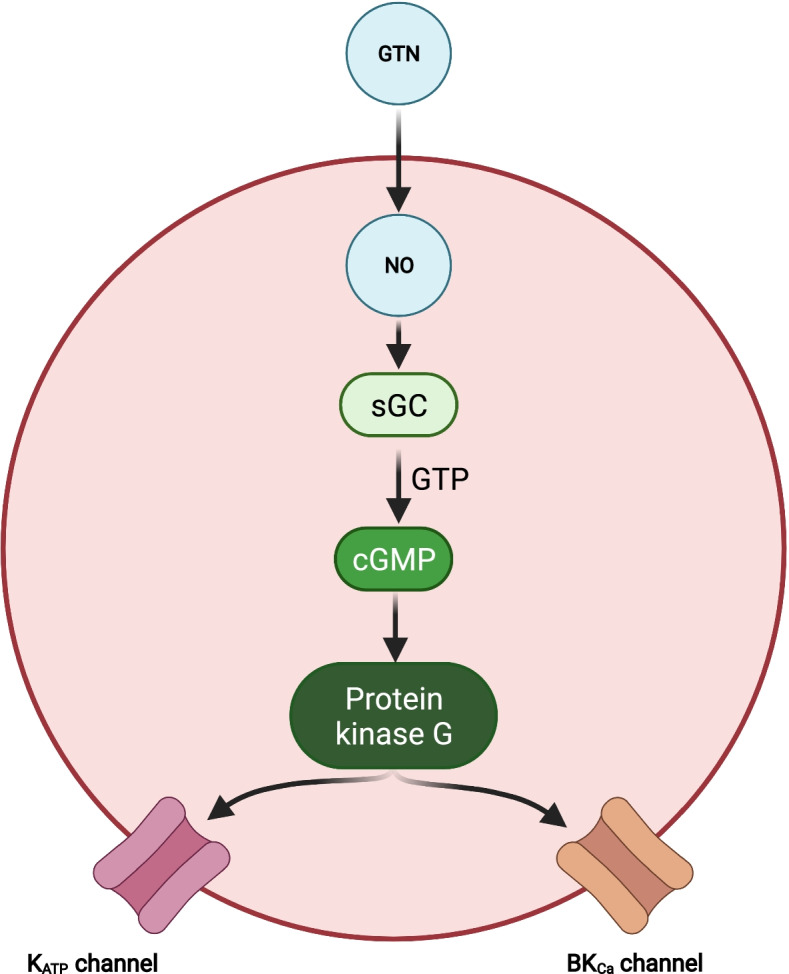
cGMP-dependent pathways in migraine pathophysiology. In vascular smooth muscle cells of the intracranial arteries, nitric oxide (NO) from glyceryl trinitrate (GTN) increases levels of cyclic guanosine monophosphate (cGMP). This activates the cGMP-dependent protein kinase (protein kinase G) which increases opening of adenosine triphosphate-sensitive potassium (K_ATP_) channels and large conductance calcium-activated potassium (BK_Ca_) channels. It will similar to the cAMP-dependent pathway ultimately activate and sensitize perivascular trigeminal afferents (see Fig. 1) [2]. BK_CA_, large conductance calcium-activated potassium channels; cGMP, cyclic guanosine monophosphate; GTN, glyceryl trinitrate; GTP, guanosine triphosphate; K_ATP_-channels, adenosine triphosphate-sensitive potassium channels; NO, nitric oxide; Protein kinase G, cGMP-dependent protein kinase; sGC, soluble guanylate cyclase

In the pathogenesis of spontaneous migraine attacks, it is known that endogenous signaling molecules are released from sensory afferents of neurons in the trigeminal ganglion and/or parasympathetic efferents of neurons in the sphenopalatine ganglion [4, 5]. These signaling molecules include adrenomedullin, amylin, calcitonin gene-related peptide (CGRP), nitric oxide (NO), pituitary adenylate cyclase-activating polypeptide (PACAP), specific prostaglandins, and vasoactive intestinal polypeptide (VIP) [6–13]. All mediate their effects on the vascular smooth muscle cells within the walls of intracranial arteries and result in opening of potassium channels and accompanying vasodilation [14, 15], (Figs. 1 and 2). This, in turn, has been hypothesized to provide the necessary mechanical and chemical stimuli to activate and sensitize the afferent nociceptive fibers that project to first order neurons in the trigeminal ganglion and upper cervical ganglia [2]. The ascending nociceptive transmission is ultimately relayed to the somatosensory cortex and other cortical areas via second order neurons in the brain stem (and their cervical extension) and third order neurons in the thalamus [2].

A key limitation of human provocation studies is that they cannot answer what causes the initial endogenous release of signaling molecules from sensory afferents and parasympathetic efferents. This step is bypassed in human provocation studies because they rely on exogenous administration of migraine-inducing substances [2]. The remaining cascade of events is, nonetheless, likely to be the same for spontaneous and provoked attacks. In support, people with migraine report themselves that the provoked attacks mimic their usual spontaneous attacks and can be effectively treated with their usual rescue medication, e.g. triptans [2, 3]. Consistent with this finding, one provocation study found that early treatment with sumatriptan was more effective in prevention of PACAP-induced migraine attacks than placebo treatment [16]. The main benefits of human provocation studies then become threefold. First, they can be used to identify signaling molecules that induce migraine attacks and are thereby implicated in migraine pathogenesis. Second, blocking the effects of the same signaling molecules might hold therapeutic promise for migraine. The advent of therapies targeting CGRP signaling seems to confirm this assertion [17]. Lastly, the combination of human provocation studies with sophisticated neuroimaging can improve our understanding of meningeal and cerebral changes during migraine attacks [3].

A research area that is ripe for improvement is the comparative assessment of provoked and spontaneous migraine attacks in the same study population. In this context, it is reasonable to assume that capturing the onset of spontaneous attacks is more feasible in chronic migraine than in episodic migraine. The pathogenic similarities and differences between provoked and spontaneous attacks can then be explored using functional and metabolic neuroimaging. Another option is to capture the onset of spontaneous attacks in women with pure menstrual or menstrually-related migraine. In this patient population, it would also be interesting to examine whether the threshold for provoked attacks differs at various time points in the menstrual cycle. An additional option that seems intuitive is to compare pathogenic mechanisms between attacks elicited by self-perceived natural triggers (e.g. stress, particular foods) and migraine-inducing substances (e.g. CGRP, PACAP). However, migraine attacks with aura were only reported by 3 (11%) of 27 participants with migraine with aura who had been exposed to their self-perceived triggers (e.g. flickering lights, strenuous exercise) [18]. This observation suggests that self-perceived triggers might be subject to false attribution and recall bias. Their use in controlled experiments is therefore questionable.

Although human provocation studies have advanced our understanding of migraine pathogenesis, new questions have emerged and should, in part, be the focus of future research efforts. There is some evidence that certain migraine-inducing signaling substances can increase the susceptibility of developing a migraine attack via a direct effect on structures within the central nervous system [19, 20]. A recent discovery was made that an opener of adenosine triphosphate-sensitive potassium channels, levcromakalim, might be a potent inducer of migraine attacks with aura [20]. Since cortical spreading depression is considered the physiological substrate of migraine aura [21], this finding suggests that levcromakalim crosses the blood–brain barrier [20]. A confirmatory study is much needed to ascertain whether levcromakalim is indeed a consistent inducer of migraine attacks with aura. If so, new avenues of research will emerge to improve our understanding of the aura-migraine linkage.

Another line of evidence suggestive of direct effects on the central nervous system relates to the NO donor glyceryl trinitrate [19]. Provocation studies have found that glyceryl trinitrate induces migraine attacks that are preceded by premonitory symptoms, i.e. non-headache symptoms that occur within minutes to hours before the onset of headache in attacks with migraine without aura [22, 23]. Premonitory symptoms are regarded as surrogate markers of activation within central nervous system structures, such as the hypothalamus [19]. Commonly reported premonitory symptoms include fatigue, neck stiffness, and mood changes [24]. These are rather vague symptoms and it is generally difficult to investigate premonitory symptoms in relation to a provoked migraine attack since most migraine-inducing substances (incl. glyceryl trinitrate) evoke a biphasic response [5]. Within minutes after the start of administration, people with migraine tend to experience an immediate mild headache that is followed by a provoked migraine attack hours later [5]. It is uncommon for the immediate headache to resolve completely before the onset of the provoked migraine attack [25]. This makes it challenging to investigate the occurrence of premonitory symptoms since they must occur before the onset of headache in a provoked attack. Taken together, data interpretation should be made with appropriate caution, and it would be ideal to establish an international consensus on the definition of premonitory symptoms in human provocation studies.

An outstanding scientific question, that merits some emphasis, is whether the induction of provoked migraine attacks depends, in part, on the duration of arterial dilation. This hypothesis has mainly been explored in human provocation studies with PACAP and VIP, both of which belong to the same family of peptides [13, 26]. In one randomized, double-blind, 2-way crossover study [26], participants with migraine were allocated to receive intravenous infusion with PACAP or VIP over 20 min on two separate experimental days. The authors found that PACAP infusion induced migraine attacks, whereas VIP infusion did not. It was also demonstrated by magnetic resonance angiography that PACAP infusion causes longer-lasting arterial dilation (> 2 h), compared with VIP infusion (< 2 h). The latter finding has typically been overlooked, whereas the former finding has often been used to conclude that arterial dilation does not have a causal role in migraine pathogenesis [27]. Nonetheless, recent provocation data have found that intravenous infusion of VIP over 120 min (instead of 20 min) causes headache and arterial dilation for > 2 h in healthy volunteers and migraine attacks in 15 (71%) of 21 participants with migraine [13]. Thus, prolonged arterial dilation cannot be disregarded as an important factor in migraine pathogenesis. Indeed, one small randomized, double-blind, placebo-controlled, 2-way crossover study found that long-lasting infusion of nitroglycerin for 420 min induces headache with migraine-like features and sustained arterial dilation in healthy volunteers who had no history of migraine or frequent headaches [28].

As migraine is a heterogeneous disease, there is also a need for research on whether different types of migraine respond the same to administration of migraine-inducing substances. This area has received little attention, although it has been established that intravenous infusion of CGRP induces migraine attacks in people with episodic migraine (with and without aura) as well as in those with chronic migraine [29–31]. It is, however, interesting that intravenous infusion of CGRP did not induce migraine attacks in people with familial hemiplegic migraine [32, 33]. Research into pathogenic differences between common and rare types of migraine might therefore be an area of interest for future human provocation studies.

In conclusion, it seems evident based on the available data that provoked and spontaneous migraine attacks are similar, except for the source of migraine-inducing substances (exogenous vs. endogenous source) and the events that precede the release of these signaling molecules from sensory afferents and/or parasympathetic efferents during spontaneous migraine attacks. Although some research questions remain unanswered, progress should be imminent with the ongoing standardization and refinement of human provocation studies.

## Data Availability

Not applicable.

## Abbreviations

CGRP: Calcitonin gene-related peptide
NO: Nitric oxide
PACAP: Pituitary adenylate cyclase activating polypeptide
VIP: Vasoactive intestinal peptide
